# Medical Gymnastics

**Published:** 1901-01

**Authors:** Theodore Toepel

**Affiliations:** Atlanta, Ga.


ATLANTA

### Journal-Record of Medicine.

Successor to Atlanta Medical and Surgical Journal, Established 1855, and Southern Medical Record, Established 1870.

VOL. II. JANUARY, 1901. No. 10.

BERNARD WOLFF, M.D., EDITOR. M. B. HUTCHINS, M.D., BUSINESS MANAGER.

Published Monthly. Office No. 64 Marietta Street, Opposite Post-office.

### ORIGINAL COMMUNICATIONS.

### MEDICAL GYMNASTICS.*

BY THEODORE TOEPEL, M.D.,
ATLANTA, GA.

I take the liberty to present to you a subject that has been recognized by the profession of the larger cities, especially the eastern, as an important adjunct to the practice of medicine and surgery. But in some way its value is less known in this section of the country than it deserves to be.

I am especially interested in this subject and hope to bring out a few points that may be of interest to you.

The title selected for my paper is "Medical Gymnastics," and the first question that every one will ask himself is, what is "Medical Gymnastics?" and this I shall endeavor to explain to you to the best of my knowledge.

*Read before the Atlanta Society of Medicine December 6, 1900.

Medical Gymnastics means systematic exercise of the muscles and other tissues for therapeutic purposes.

Medical Gymnastics has been commonly known as a "Movement Cure" "Movement Treatment," and whereas some authors cannot lay enough stress on the distinction between Medical Gymnastics and Massage, others have included under the last name all forms of manual therapeutics. Massage, however, is usually understood as denoting one of the procedures of friction, kneading, pressure and percussion, each one of which may be regarded as passive exercise of the muscles.

It will then be most convenient to include Massage in the name "Medical Gymnastics," all the more since a skilled masseur never relies exclusively on massage proper, nor a medical gymnast on movements.

### POSITION AND MOVEMENT.

A movement is a change from one position to another, and might be said to consist of a sequence of positions. In order to accomplish a definite, preconceived purpose, commencing position, final position, sphere of action, velocity, force and duration must be exact.

### EFFECTS OF MOVEMENTS.

"The effect of a movement is the result of its action, and the reaction of our organism" (Roth). The effect may be physical, physiological and psychological, and movements also have both a general and a local effect. While the local effect usually decides the choice of a movement, its general effects should never be left entirely out of consideration.

Medical Gymnastics should be applied as local or as constitutional treatment in accordance with these effects.

The general effects of active movements are those of ordinary gymnastics, a general increase of the functional activity; resistive movements emphasize these effects, while assistive movements produce them in a less degree. Active movements involve the use of attention, and consequently cultivate voluntary control, while passive movements diminish motor irritability and voluntary coordination, and favor inattention. Active movements diminish sensory, and heighten motor irritability, harden the fibrous tissues, and lessen the adipose ones; passive movements may increase sensory and lessen motor irritability, make lamellous texture predominant and cause a greater amount of fat to accumulate between the muscles and under the skin.

A movement is active when executed by the patient himself; if he does it alone it is called single, if the operator helps him in performing it, it becomes assistive; if one, or the other, or a machine offers resistance, resistive. These movements are so related in progression that the assistive precede the others for reasons plain to every one; and for a majority of cases the resistive precede the single, for the reason that in resistive movements the launching effort is partly reflex, caused by the contact of the apparatus or the operator's hand; so that the single movement really requires greater cerebral impulse—is more difficult from a neurological point of view.

Active movements begin and end in the brain, so that their first effect will be the increase of mental activity with consequent greater afflux to the active centers. The constant use of motor nerves will increase the irritability of these and it will be found that sensory nerves meanwhile become less impressionable. For that reason active movements are most decidedly indicated for "nervous" invalids who are not weak enough for massage.

Active movements presuppose attention, will, and coordination, and consquently develop these, while passive movements favor inattention, relaxation. Active exercises also produce a consciousness of power, that ability to do (the aggregate of muscular sense) which in so many cases is a long step toward a cure, and which cannot be gotten by purely passive movements.

Active movements increase the heart-beat. The general nutritive activity is heightened, since nutritive material is carried more quickly to the tissues, also because the increased pressure causes increase of osmotic phenomena.

Assimilation and elimination increase so that the general metabolism is much heightened, raising the standard of general bodily health and strength.

Locally active movements cause an increased afflux to the parts put into motion, and there will be a consequent increase of local nutritive activity, growth of muscle and bone is encouraged, and even the fibrils of the active nerve increase in number and size. The active movements, then, become a means of developing undersized, atrophied, and paretic muscles, and of bringing them under the control of the will, in this respect far superior to the passive movements, since these do not increase muscular contractility or motor neurition. The passive movements merely produce and maintain the power of existing, and so are in reality mere stepping-stones to higher ends, a means of tiding the weakling over, and preparing him for a more normal type of activity.

Active movements then must be the final aim in medical gymnastics; and massage is to be considered merely as an introductory measure, and as an auxiliary when the subject is considered as a whole. This, of course, does not lessen the merit of massage as a specific application in a great many diseases, where in the nature of things active movements would be contraindicated.

The resistive movements are usually done so that the patient executes the movement while the operator resists, but may in special cases be reversed, the patient offering resistance to the operator's movement. This form is especially useful in cases of motor paralysis where the voluntary power is just returning. The resistance offered should be slight at the beginning and the end of the movement, since the muscles have the poorest levers at these points. Toward the middle of the movement the lever grows longer, and the resistance should increase proportionately. It is customary also to begin and end each movement with passive extension, it being found that a muscle contracts more readily if it is stretched first. This is due to the fact that the recoil then assists the contractions, so that in reality the beginning of the latter is passive. Besides, passive extension has the effect of hastening the venous current, and in that manner the fatigue products being carried away more quickly, the endurance in the muscle increases, *i. e.*, the resistive movement so prepared will give all the beneficial results and hardly any of the effects of fatigue. The muscles may also be prepared for contraction by massage, since this has been found to more than double their power of endurance. The resistance offered must be free from tremor, pushes, or starts. The grasp should be light and easy.

GENERAL PHYSIOLOGICAL EFFECTS OF EXERCISE.

As Dr. Lagrange well expresses it, "Exercise produces in the system two absolutely different effects: it increases the process of assimilation, thanks to which the body gains new tissues, and it accelerates the process of dissimilation, which leads to the destruction of certain materials." Its action in the former direction depends upon the increased amount of oxygen introduced into the system by the improved circulation and respiration, and by the healthy stimulation of the various active organs of the body.

The need for exercise is felt as much by thin people, who assimilate too little, as by fat people who do not dissimilate enough. Exercise may therefore be regarded as a great regulator of nutrition.

As the action of the heart rapidly increases in force and frequency during exercise, the flow of blood through all parts of the body is increased. The amount of increase is from ten to thirty beats, but it may be more. The skin becomes red with the blood contained in the full capillaries, and perspiration is much increased. The amount of fluid which is lost by the skin is very considerable.

The digestive apparatus is stimulated and strengthened by exercise. The appetite improves, digestion is more complete, absorption more rapid, and the circulation through the liver is more vigorous and even.

Muscular exercises, especially such as employ the muscles of the abdomen, have a very beneficial effect upon the bowels, promoting peristaltic movements and relieving such constipations as depends upon the torpidity of the intestine. Moderate, regular, and systematic exercise by stimulating the circulation of the body improves also the circulation of the brain, and is therefore an aid to cerebral movements. It improves the health and the physical strength, and so increases the capability of the individual for mental work and for the physical strain incident upon mental concentration.

By organizing in the brain a series of muscular movements, by elaborating the powers of coordination, and by establishing automatism in a large and varied series of actions, it saves actual brain work and renders a considerable number of movements independent of the direct action of the will.

It offers, too, an admirable change of employment. There is no better rest from severe mental work than well selected bodily exercises.

### DISEASES OF THE CIRCULATORY ORGANS.

The experience that diseases in the organs of circulation are favorably influenced by medical gymnastics has, ever since its introduction, been more and more acknowledged. This fact has also been gradually admitted, that disorders, not only in the function of the heart, but also those in the more peripheral parts of the apparatus of circulation, in the vessels and capillaries, experience a beneficial influence from medical gymnastics. It is frequently necessary, of course, that the treatment continue a long time and be often repeated, but this will be the case, too, with all other remedies for heart diseases.

A weakened circulation is the necessary consequence of every heart disease, and the object of medical gymnastics will therefore be to facilitate the work of the heart by improving the weakened circulation. This best takes place in most cases by diminishing the congestion in the venous system, and there are three large groups of passive movements that have a direct circulatory-furthering effect, namely: Kneadings, rollings and respiratory movements.

Muscle kneadings and rollings further the circulation, especially in the more peripheral parts of the body; respiratory movements do the same in the chest, and, indirectly, in the abdominal cavity also, for which the abdominal kneading is specially of importance on account of venous congestion in the digestive apparatus.

### DISEASES OF THE RESPIRATORY ORGANS.

Educational gymnastics is, in itself, of importance as a prophylactic medium in most diseases of the chest, and it powerfully supports other prophylactic treatment. Medical gymnastics is, in a still higher degree than educational, of prophylactic importance in diseases of the chest, because it can more directly be arranged so as to expand a sunken-in chest, make a shallow respiration normal and the interchange of air as complete as possible; at the same time the respiratory muscles become strengthened through the treatment.

### DISEASES OF THE DIGESTIVE ORGANS.

Of stomach and intestinal diseases only the more chronic forms have hitherto been treated with medical gymnastics. This is also the case, for that matter, with most other illnesses, but in diseases of the digestive organs it is very seldom that medical gymnastics is used until all other remedies have previously been tried with more or less success during a period of many years; and still the purely mechanical treatment in most cases offers more and greater advantages than any other. The direct manipulation of the abdomen causes more lively secretion from the mucous surface of the stomach and intestines; it increases the intestinal power of re-absorption, and it should moreover help, as in other parts of the organism, to remove an unhealthy formation of mucus from the surface of the mucous membrane; a better and more complete mixture in the digestive canal of the food substances and the secreted juices is also gained, and finally a more even and regular purgation is obtained.

The importance of medical gymnastics in the treatment of stomach and intestinal affections can be said in most cases only to support other treatment than to act alone.

### DISEASES OF THE NERVOUS SYSTEM.

Medical gymnastics, at the present time, has gained its greatest success and seen the greatest of its newly acquired results within the branch of diseases of the nervous system. If a medical gymnastic treatment, in a great number of cases, has been of little or no good, it has then only been one with all other remedies used, but it has, on the other hand proved beneficial and produced essential improvement in some diseases of the nervous system, which have before been declared inaccessible to treatment.

The most important thing in all treatment is just to set a proper diagnosis. Specialists ought to be consulted, when the diagnosis is uncertain. Great benefits have been derived from its use in peripheral nervous diseases, vasomotor and trophic neurosis, diseases of the spinal cord, neurosis without any known anatomical causes.

DISEASES OF THE MUSCLES.

Atrophy, contracture and muscle inflammation are the muscle diseases which are generally treated with gymnastics. Muscle kneading is here, perhaps, the most important movement. Direct examinations have proved that massage, and especially muscle kneading, given during 2–5 m. doubles the unit of work which the healthy muscle can perform. On all occasions, when the muscle is abnormally shortened or stretched, as in diseases of the osseous system with deformity of any part of the skeleton, after external injuries and surgical operations, in muscular contractions after paralysis, etc., the gymnastic movements are as important as the muscle kneading.

CONSTITUTIONAL DISEASES.

Under this head are generally included diseases of the blood and blood-forming organs and diseases which have their origin in a disturbed metabolism. In the former group are included anemia, chlorosis, hemophilia, etc.; in the latter are included fatty degeneration, gout, diabetes, rickets, etc.

A generally strengthening treatment is necessary in the above mentioned diseases, as well as in many other illnesses, but often, even when no special illness exists, diet, spring and bath cures, strong food and strengthening medicine are the most general ordinations, and in most cases they are sufficient; but gymnastics is even here of great importance, in some cases, perhaps, mostly as supporting the effect of the above mentioned means, but in others as the remedy which is the most beneficial.

Constitutional diseases often depend on too little physical exercise in general, on bad appetite and bad utilization of the food taken, and the consequently disturbed metabolism and lack of blood formation. That medical gymnastics, better than any other means, compensates for the muscle and physical work necessary to the well-being of the body has been pointed out before. Through the muscle movements the nourishment taken into the body is consumed and best turned to account, and, at the same time, the necessity for an increase of nourishment is produced; the circulation of the blood will be more active, the quantity of blood will be increased, and, at the same time, an increase in all the tissues and organs of the body will arise.

No speedy results can be expected from medical gymnastics, as little in constitutional diseases as in local. The effect of gymnastics on the contrary comes slowly, but it will be of longer duration than that of many other remedies.

### DISEASES OF THE BONES AND JOINTS.

In the gymnastic treatment of a joint, it is of the greatest importance to know the physiological limits for every one of its movements. This holds good for gymnastic movements in the joints generally, but it is specially of importance that the normal limits be not exceeded in the treatment of a swollen or stiff joint, which easily happens in the attempt to gain the greatest possible mobility in the joint.

In the treatment of the joints the necessity for a careful choice between massage and gymnastics shows itself more than otherwise. In an acute synovitis, in a swelling in and around a joint after a distortion, a massage treatment can be sufficient to give restored functional capacity, but whenever the object is to overcome a stiffness in the joint—for example, after a dislocation, a more considerable contusion or a rheumatoid arthritis, which are the commonest causes of stiffness, then the gymnastic movements are as important as massage, and the more so the longer the stiffness has existed. If some time has elapsed between the occurrence of the injury and the commencement of the treatment, then massage alone cannot accomplish much, but an energetic movement treatment becomes necessary. The movements must often be continued with long after the swelling, ache and pain in the joint have subsided, that is, after the massage is superfluous.

The best results are gained, therefore, if from the beginning of the treatment massage and gymnastics are given in a proper combination, after which gymnastics is continued until perfectly good results are attained.

SPINAL CURVATURES.

Spinal curvatures are reasonably included amongst the more serious of the acquired deformities that occur in the growing generations, because they so frequently leave unpleasant consequences for the whole life. It is, therefore, of great importance that they be discovered in time and suitably treated as early as possible, and a spinal curvature can easily be detected by every physician, if he only takes the trouble to look at the naked backs of the children.

The exercises for the correction of this trouble should be such as develop the muscles of the back, including the neck; the glutei muscles and others about the hip usually also need exercising, and the abdominal muscles also, in most cases.

DIETETIC GYMNASTICS.

In connection with the accentuation of the importance of gymnastics in constitutional illnesses, it has also been said that it can be used without there existing any real disease. Medical gymnastics is used under the last mentioned conditions principally for those persons, who, from one cause or another, get too little bodily movement, such as, for instance, after long confinement to bed in slow convalescence, for weakly developed youths, for very old persons, and also for persons, who in the best years of their active life, are hindered from taking necessary exercise. The necessity and use of a gymnastic treatment, under the above-mentioned circumstances, ought to be fully clear from what has already been said when speaking of constitutional illnesses.

Very often the observation must also be made, that young people, who have received gymnastic treatment for a spinal curvature or for some other local complaint, at the same time that they have had the real disease cured, have also gained a better appetite, strengthened muscles, and a stronger development in general, besides which headache, general listlessness and weariness and other nervous symptoms have at the same time disappeared.

Gymnastics is and will remain one of the best remedies of medicine, for it can cause a weakened circulation to be improved, a superficial respiration to be deeper and fuller; a disturbed digestion to be restored; paralyzed, and weak muscles to become functionally capable; physical deformities to be checked, the nervous system to experience a soothing influence, and finally both weakly developed adolescence and old age, worn-out with work, to gain an increased vitality.

